# Factors associated with Legionnaires’ disease recurrence in hotel and holiday rental accommodation sites

**DOI:** 10.2807/1560-7917.ES.2019.24.20.1800295

**Published:** 2019-05-16

**Authors:** Julien Beauté, Sven Sandin, Birgitta de Jong, Lara Payne Hallström, Emmanuel Robesyn, Johan Giesecke, Pär Sparén, on behalf of the European Legionnaires’ Disease Surveillance Network

**Affiliations:** 1 European Centre for Disease Prevention and Control (ECDC), Stockholm, Sweden; 2 Department of Medical Epidemiology and Biostatistics, Karolinska Institutet, Stockholm, Sweden; 3 Department of Psychiatry, Icahn School of Medicine at Mount Sinai, New York, United States; 4 Seaver Autism Center for Research and Treatment, Icahn School of Medicine at Mount Sinai, New York, United States; 5 Department of Public Health Sciences, Karolinska Institutet, Sweden; 6 Members of the European Legionnaires’ Disease Surveillance Network are acknowledged at the end of the article

**Keywords:** Legionnaires’ disease, travel, surveillance, waterborne infections, Legionella, Europe, bacterial infections, surveillance, epidemiology

## Abstract

**Background:**

The detection of a cluster of travel-associated Legionnaires’ disease (TALD) cases in any European Union/European Economic Area (EU/EEA) country prompts action at the accommodation, follow-up by health authorities and reporting of measures taken. Some accommodations incur further cases despite presumed implementation of adequate control measures.

**Aim:**

To identify factors associated with the occurrence of a further TALD case after the implementation of control measures.

**Methods:**

We conducted a retrospective cohort study of hotel and holiday rental accommodations in the EU/EEA associated with two or more TALD cases with onset dates less than 2 years apart (a ‘cluster’) and notification between 1 June 2011−31 December 2016. We fitted Cox regression models to estimate the association between accommodation characteristics and the occurrence of a further case, defined as any case with onset date after the report on measures taken.

**Results:**

Of the 357 accommodations in the analysis, 90 (25%) were associated with at least one further case after the report on measures taken (12.4/100 accommodation-years). Accommodations associated with two or more cases before the cluster notification were more likely to be associated with a further case, compared with those not previously associated with any case (adjusted hazard ratio 1.85; 95% confidence interval: 1.14–3.02). Neither the detection of *Legionella* in the water system nor the type of disinfection were found to be associated with the risk of a further case.

**Conclusion:**

Accommodation size and previous TALD cases were predictive of further Legionnaires’ disease cases after implementation of control measures.

## Background

Legionnaires’ disease (LD) is a severe pneumonia caused by Gram-negative bacteria, *Legionella* spp., which develop in aquatic environments and can contaminate man-made water systems [1]. People are infected by inhaling contaminated aerosols; person-to-person transmission has only been described once [2]. LD is notifiable in all 31 European Union/European Economic Area (EU/EEA) countries, where ca 70% of all reported cases are community acquired, 20% travel associated and 10% healthcare related [3].

Travel-associated infection has played a central role in LD history since its first description during a large outbreak among members of a United States (US) organisation of war veterans attending a convention at a hotel in Philadelphia in 1976 [4]. Three years after the identification of the causative pathogen, a retrospective analysis of pneumonia cases in travellers to Benidorm, Spain in 1973 suggested that several cases were possibly LD cases associated with the same hotel over a period of several years [5]. Travel is a known risk factor of LD for a variety of reasons. First, hotels and similar accommodation sites often have complex water systems with a large number of outlets (e.g. showers) [6]. In the absence of regular flushing (e.g. an unoccupied room or annual closure) the water may stagnate in the pipes, favouring the growth of *Legionella*. In addition, it can be difficult to maintain adequate temperature control of hot and cold water with long pipe systems. Second, these accommodation sites are likely to have facilities known to be associated with an increased risk of LD, such as whirlpool spas [7]. Last, hotels may host a large number of visitors, who may be exposed to the same source during their stay.

A travel-associated Legionnaires’ disease (TALD) surveillance system at the EU/EEA level has been in place since 1987 [8] and has been part of the European Legionnaires’ disease surveillance network (ELDSNet) since 2010. For 2011–15, EU/EEA countries reported 750–1,100 TALD cases annually [9-13]. ELDSNet detected ca 80–160 new clusters per year (two or more TALD cases with onset dates less than 2 years apart), of which 50–60% would most likely not have been detected without international collaboration.

The detection of a cluster of TALD in any EU/EEA country prompts action at the accommodation and follow-up by health authorities, with a summary of measures taken reported to ELDSNet. Accommodations that fail to satisfactorily implement the recommendations made by the competent authorities are listed on the European Centre for Disease Prevention and Control (ECDC) website [14]. Despite adequately following recommendations, some facilities incur further cases for reasons that are not fully understood. A previous study suggested that ca 20% of the accommodation sites investigated between 2003–07 could be associated with a further case [6].

Understanding the optimum methods for environmental control has been listed as one of the research priorities for LD [7]. Therefore, the objective of this study was to identify factors associated with the occurrence of further cases after implementation of control measures to improve prevention and control of TALD.

## Methods

### Data sources

Nominated experts from 31 EU/EEA countries report TALD cases that meet the EU case definition [15] to ELDSNet on a daily basis, with a set of variables including main demographics, laboratory data and travel history of the case [13]. According to the ELDSNet definition, a cluster consists of two or more TALD cases with onset dates less than 2 years apart, who stayed in the same accommodation site during the 2–10 days before onset of disease. A ‘complex cluster’ is defined as two or more clusters with at least one case in common. ELDSNet notifies national public health authorities of each cluster, who then investigate the accommodation site involved. These authorities report their early risk assessment 2 weeks after notification (through a so-called ‘form A’) and provide the final results of environmental sampling and control measures 6 weeks after notification (form B). For the purpose of this analysis, we used the information collected in this final report (form B), including detection of *Legionella* in the water system, presence of preventive measures before the cluster (until 2015), type of disinfection and overall assessment of control measures [14]. The form B does not collect information on the laboratory method used to detect *Legionella* in the environmental specimens. We also used some of the accommodation site characteristics, including accommodation type (e.g. hotel) and location (country). Since large hotels host larger numbers of travellers, they may have a higher probability of being associated with subsequent cases [16]. To control for the accommodation size, we estimated the number of rooms for each accommodation site through popular travel website companies (Booking.com and TripAdvisor).

### Inclusion and exclusion criteria

We included all hotel and holiday rental accommodation sites in the EU/EEA that were associated with a cluster of TALD cases notified between 1 June 2011−31 December 2016. We selected this start date because the Epidemic Intelligence Information System for ELDSNet (EPIS-ELDSNet) started in May 2011. EPIS-ELDSNet is a web-based communication platform that allows nominated public health experts to exchange technical information on LD, with a focus on the detection and follow-up of travel-associated clusters. We excluded campsites (e.g. tent, mobile home, caravan, etc.) because they encompass heterogeneous living spaces of a different character than hotels and holiday rental accommodations, which use different types of water outlets. In addition, it is difficult to obtain a reliable proxy for the size of these sites. We excluded complex clusters from this analysis because the link between the accommodation site and the cluster is weaker. To explore possible reports of previous cases, we included all cases reported since the late 1980s [8].

### Scenarios

**Figure 1:**
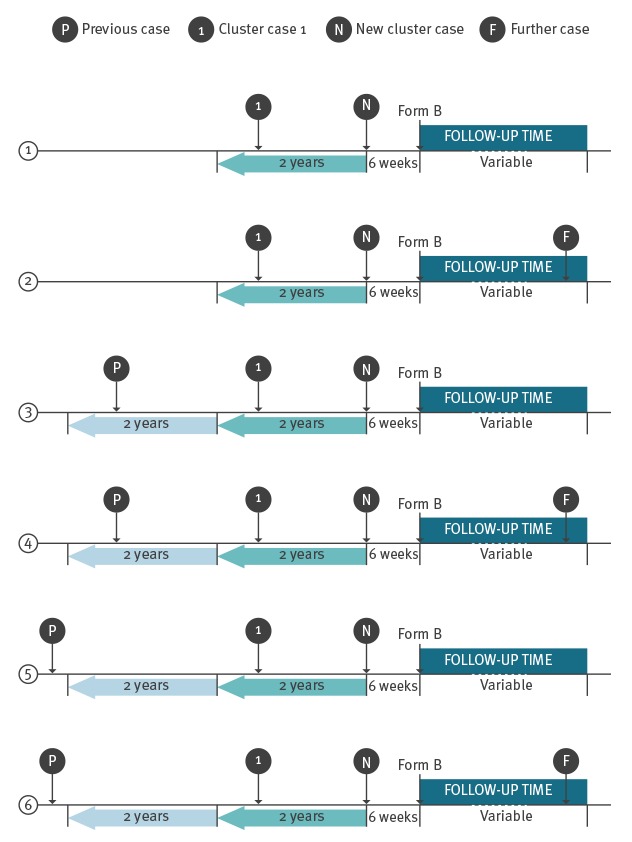
Schema of the six scenarios considered in the analysis of factors associated with Legionnaires’ disease recurrence in hotel and holiday rental accommodation sites, EU/EEA, 2011–2016

The main outcome in this study was the occurrence of a further case, which we defined as any case with onset date after the report on measures taken (form B). For the purpose of this analysis, we considered six scenarios (Table 1 and Figure 1). In scenarios one and two there was no event (report of a previous case or a cluster) before the cluster under consideration. In scenario two the accommodation was reported with a further case (or cluster) after receipt of form B, but not in scenario one. In scenarios three to six there was an event (report of a previous case or cluster) before the cluster under consideration. In scenarios three and four, the most recent previous case and the first case of the cluster under consideration had onset dates less than 2 years apart. Technically, this means that the previous case and at least the first case of the cluster under consideration formed a first cluster. Because of the occurrence of a later case with onset date more than 2 years after the onset date of the previous case, the latter case fell out of the cluster and was not part of the cluster under consideration. However, it is possible that authorities investigated the site, implemented control measures and reported on them (form B). In scenarios five and six, the previous case and the first case of the current cluster had onset dates more than 2 years apart. In scenarios four and six the accommodation was associated with a further case (or cluster) after receipt of form B.

**Table 1:** Summary of the six scenarios considered in the analysis of factors associated with Legionnaires’ disease recurrence in hotel and holiday rental accommodation sites, EU/EEA, 2011–2016

Scenario	Report of a previous case or a cluster		Report of a further case or cluster
	< 2 years before first case of cluster	≥ 2 years before first case of cluster	
1	N	N	N
2	N	N	Y
3	Y	N	N
4	Y	N	Y
5	N	Y	N
6	N	Y	Y

EU/EEA: European Union/European Economic Area; N: no; Y: yes.

EU/EEA: European Union/European Economic Area.

A ‘form B’ is a summary report of the final results of environmental sampling and control measures, 6 weeks after notification.

### Statistical analyses

We compared the main characteristics and results of environmental sampling and control measures for accommodation sites reporting a further case. Categorical variables were compared using a chi-square test. Continuous variables were compared across strata with the Mann–Whitney U test. We calculated the rate of occurrence of a further case over the study period and the corresponding two-sided 95% confidence interval (CI). We compared survival functions using the log-rank test. We censored accommodation sites without any subsequent case on 31 December 2016 (right censoring). We fitted Cox regression models and calculated hazard ratios (HR) with associated two-sided likelihood ratio type tests and 95% CI to estimate the association between accommodation sites’ characteristics and the occurrence of a further case, using time since form B as the primary time scale. We first fitted the Cox models including each variable of interest separately (accommodation characteristics and information collected in the final report (form B)). We then ran an adjusted model including all variables with statistically significant association in the univariate analysis (95% CI, excluding the null value). We examined the proportional hazards assumption on the basis of plots of Schoenfeld residuals [17].

We classified the number of rooms in four categories according to the overall quartile distribution. We classified accommodations according to their possible association with a previous case in three categories (no previous case, one case, two or more cases). In the main analysis we did not distinguish accommodations with a previous case according to the time between onset dates of the previous case and the first case of the cluster.

We performed sensitivity analyses: (i) excluding scenarios three and four, because the report of measures taken for accommodations sites under these scenarios may not be the first one for the included cluster and (ii) excluding all accommodations with a previous case (scenarios three to six), because these accommodations may have a long history of recurrent cases and may therefore be particularly difficult to remediate.

We used Stata software release 14 (StataCorp. LP, United States) for all statistical analyses.

## Results

### Characteristics of the accommodations sites

Of the 395 accommodation sites in the EU/EEA with a cluster notified between 1 June 2011−31 December 2016, 357 (90.4%) had available information on both follow-up (form B) and number of rooms. Of these 357 accommodation sites, 339 (95.0%) were hotels, 10 (2.8%) were apartments and 8 (2.2%) were an ‘other’ accommodation type (e.g. aparthotel) (Table 2). The median number of rooms was 68 (interquartile range (IQR): 36–127). These accommodations were located in 21 EU/EEA countries. The countries with the highest proportions of accommodations associated with a cluster during the specified period were Italy (42.6% of all accommodation sites), Spain (17.1%), France (14.6%) and Greece (7.6%).

**Table 2:** Frequency, distribution and rates of first further travel-associated Legionnaires’ disease (TALD) case after control measures in hotel and holiday rental accommodation sites associated with a cluster of TALD, EU/EEA, 1 June 2011–31 December 2016

Characteristics	All accommodations		Accommodations with further cases		Rate of further cases per 100 accommodation-years		
	n	%	n	%	n	95% CI	p value^a^
Total	357	100	90	100	12.4	10.1–15.2	–
**Accommodation type**							
Hotel	339	95.0	87	96.7	12.7	10.3–15.7	0.536
Apartment	10	2.8	2	2.2	7.6	1.9–30.2	
Other	8	2.2	1	1.1	5.4	0.8–38.5	
**Number of rooms**							
0–35	86	24.1	12	13.3	6.1	3.5–10.8	0.043
36–67	91	25.5	26	28.9	14.6	10.0–21.5	
68–126	90	25.2	28	31.1	15.1	10.4–21.8	
127–775	90	25.2	24	26.7	14.3	9.6–21.3	
**Country of travel**							
France	52	14.6	12	13.3	10.7	6.1–18.8	0.170
Greece	27	7.6	6	6.7	11.2	5.0–24.9	
Italy	152	42.6	44	48.9	15.1	11.2–20.3	
Spain	61	17.1	19	21.1	14.8	9.4–23.2	
Others	65	18.2	9	10.0	6.4	3.3–12.2	
**Previous notifications**							
None	227	63.6	45	50.0	9.4	7.0–12.6	0.002
One case	61	17.1	18	20.0	14.6	9.2–23.2	
≥ 2 cases	69	19.3	27	30.0	21.3	14.6–31.1	
**Cluster size**							
Two cases	296	82.9	71	78.9	12.1	9.6–15.2	0.548
≥ 3 cases	61	17.1	19	21.1	13.6	8.7–21.3	
**Six week post-cluster report**							
**Preventive measures in place before the cluster**							
Yes	237	66.4	71	78.9	12.4	9.8–15.6	0.952
No	37	10.4	11	12.2	12.7	7.0–22.9	
Unknown	83	23.2	8	8.9	11.9	6.0–23.8	
***Legionella* found in water system**							
Yes	229	64.1	59	65.6	12.6	9.8–16.3	0.859
No	111	31.1	28	31.1	12.3	8.5–17.8	
Unknown	17	4.8	3	3.3	9.1	2.9–28.1	
**Disinfection**							
Thermal and chemical	157	44.0	45	50.0	14.6	10.9–19.6	0.669
Thermal	53	14.8	13	14.4	11.7	6.8–20.2	
Chemical	52	14.6	13	14.4	12.1	7.0–20.8	
No disinfection	26	7.3	5	5.6	9.1	3.8–21.9	
Unknown	69	19.3	14	15.6	9.6	5.7–16.2	
**Satisfactory control measures**							
Yes	293	82.1	75	83.3	12.9	10.3–16.1	0.868
No	35	9.8	7	7.8	10.5	5.0–22.0	
Unknown	29	8.1	8	8.9	10.4	5.2–20.7	

CI: confidence interval; EU/EEA: European Union/European Economic Area.

^a^ p value of log-rank test for equality of survival functions.

### Cluster size and scenarios

The average cluster size notified by the 357 analysed accommodations was 2.3 TALD cases (range: 2–15); 296 (82.9%) accommodations had two cases and 61 (17.1%) had three cases or more. In addition, 227 (63.6%) had never been associated with any case before the cluster (scenarios one and two) and 130 (36.4%) had previously been associated with at least one TALD case (scenarios three to six), on average 2.4 cases (range: 1–11) (Table 2). Of the 130 accommodation sites associated with a previous case, 120 (92.3%) had their most recent previous case with an onset date more than 2 years before the first case of the cluster (scenarios five and six) and 10 (7.7%) had onset dates less than 2 years apart (scenarios three and four). After receipt of the six-week post-cluster report (form B), 90 (25.2%) of the 357 accommodation sites were associated with at least one further case (scenarios two, four and six), with on average 2.1 further cases (range: 1–22).

### Report on environmental investigation and control measures

*Legionella* was detected in the water system of 229 (67.4%) of the 340 accommodation sites for which the results of environmental investigation were available. In 237 (86.5%) of the 274 accommodations sites with this information available, preventive measures were in place before the investigation. Of the 288 accommodation sites with available information on disinfection, 157 (54.5%) had their water system treated by both thermal and chemical disinfection in response to the cluster, 53 (18.4%) by thermal disinfection alone, 52 (18.1%) by chemical disinfection alone and 26 (9.0%) were not disinfected. Of the 26 accommodations that were not disinfected in response to the cluster, 18 had prevention measures in place, two did not and the remaining six had no information available. Overall, the national authorities concluded that measures taken were satisfactory for 293 (89.3%) of the 328 accommodations for which this information was available.

### Accommodations associated with further cases

Ninety (25%) accommodation sites were associated with at least one further case a median time of 304 days (IQR: 166–610) after the report on measures (form B). Compared to those not associated with any subsequent cases, accommodations associated with one or more further cases after the cluster had a large number of rooms (median 79 vs 62 rooms; p = 0.03) and were more likely to have been previously associated with one or more TALD case before the cluster (50.0% vs 31.8%; p < 0.01). For other characteristics, there was no difference between accommodation sites according to their association with a further case (Table 2).

### Survival analysis

**Figure 2:**
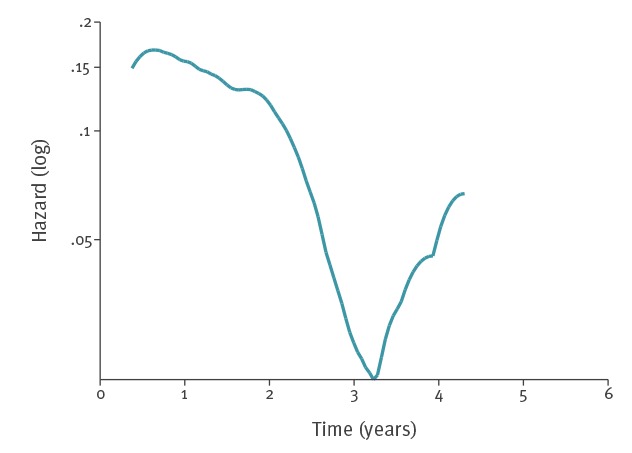
Smoothed hazard function of occurrence of further TALD cases after control measures in hotel and holiday rental accommodation sites, EU/EEA, 1 June 2011–31 December 2016

**Figure 3:**
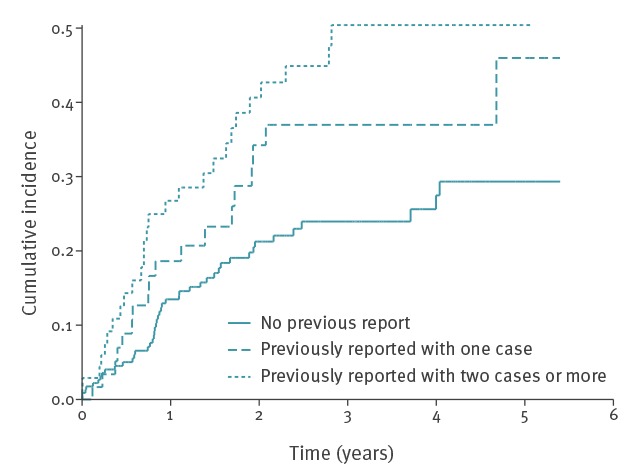
Cumulative incidence of hotel and holiday rental accommodations sites associated with a further TALD case after control measures, by previous report status, EU/EEA, 1 June 2011–31 December 2016

The 357 accommodation sites included in the analysis had an average follow-up time of 2 years, ranging from 1 day to 5 years and 4 months. The overall rate of occurrence of further cases after the report on measures was 12.4 per 100 accommodation-years (95% CI: 10.1–15.2) (Table 2). We observed the highest hazard rates (> 15/100 accommodation-years) during the first 2 years of follow-up. The hazard function decreased to a virtually null rate during the third year and then increased in the following years to five per 100 accommodation-years (Figure 2). After 1 year of follow-up, 17% of the accommodation sites (95% CI: 13–21%) were associated with a further case. This proportion was 28% (95% CI: 23–33%) after 2 years and 34% (95% CI: 28–40%) after 4 years. Cumulative incidences of accommodation sites associated with a previous case were higher compared with those that were never associated with any case before the cluster (p < 0.01) (Figure 3). After 4 years of follow-up, 29% (95% CI: 22–39%) of the accommodations were associated with a further case. This proportion reached 44% (95% CI: 35–55%) for those associated with at least one case before their inclusion in this study (p < 0.01).

EU/EEA: European Union/European Economic Area; TALD: travel-associated Legionnaires’ disease.

EU/EEA: European Union/European Economic Area; TALD: travel-associated Legionnaires’ disease.

The risk of a further LD case was higher in accommodation sites with 36–67 rooms, compared to those with < 36 rooms, with an estimated HR of 2.34 (95% CI: 1.18–4.64) (Table 3). Accommodations with more than 67 rooms had the same risk as accommodations with 36–67 rooms. Accommodations previously associated with two cases or more before the cluster had a statistically significant higher risk of a further case compared with those that were not (HR = 2.26; 95% CI: 1.40–3.64). Neither the detection of *Legionella* in the water system nor the type of disinfection were found to be associated with the risk of a further case. Cluster size was not associated with the risk of subsequent cases. In the multivariable analysis, accommodations previously associated with two cases or more before the cluster had a statistically significant higher risk of occurrence of a further case compared with those without previous cases (adjusted HR: 1.85; 95% CI: 1.14–3.02). There was no evidence for non-proportional hazards.

**Table 3:** Univariate and multivariable Cox proportional hazards models of the association between hotel and holiday rental accommodation sites’ characteristics and occurrence of a further TALD case after control measures, EU/EEA, 1 June 2011–31 December 2016

Risk factors	Univariate analysis			Multivariable analysis^a^		
	Hazard ratio	95% CI		Hazard ratio	95% CI	
**Number of rooms**						
0–35	ref			ref		
36–67	2.34	1.18	4.64	2.29	1.13	4.64
68–126	2.45	1.25	4.83	2.31	1.16	4.62
127–775	2.22	1.11	4.45	2.27	1.09	4.72
**Previous case before cluster**						
None	ref			ref		
One case	1.58	0.92	2.74	1.56	0.90	2.70
≥ 2 cases	2.26	1.40	3.64	1.85	1.14	3.02
**Cluster size**						
Two cases	ref			NA		
≥ 3 cases	1.17	0.70	1.94	NA		
**Six week post-cluster report**						
**Preventive measures in place before the cluster**						
Yes	ref			NA		
No	0.97	0.52	1.84	NA		
Unknown	0.89	0.42	1.87	NA		
***Legionella* found in water system**						
Yes	ref			NA		
No	0.99	0.63	1.56	NA		
Unknown	0.72	0.23	2.31	NA		
**Thermal disinfection**						
Yes	ref			NA		
No	0.76	0.43	1.34	NA		
Unknown	0.78	0.46	1.34	NA		
**Chemical disinfection**						
Yes	ref			NA		
No	0.80	0.46	1.37	NA		
Unknown	0.69	0.39	1.22	NA		
**Satisfactory control measures**						
Yes	ref			NA		
No	0.84	0.39	1.83	NA		
Unknown	0.88	0.42	1.82	NA		

CI: confidence interval; EU/EEA: European Union/European Economic Area; NA: not available; ref: reference category; TALD: travel-associated Legionnaires’ disease.

^a^ Adjusted for country of travel.

The sensitivity analysis excluding scenarios three and four yielded results that were similar to the main analysis. When restricting the analysis to the 227 accommodations that were never associated with any case before the cluster (scenarios one and two), a large cluster size at notification (> 2 cases) was associated with a lower risk of being associated with a further case compared with small clusters (2 cases) with an adjusted HR of 0.31 (95% CI: 0.10–0.93).

## Discussion

Our results suggest that 28% of the accommodation sites associated with clusters of TALD cases were associated with at least one further case within 2 years, despite implementation of presumably effective control measures. We observed the highest risk for occurrence of a further case in the first 2 years after receipt of the final report on control measures (form B). Among the factors examined for a potential association with further TALD cases, accommodation size and multiple earlier LD cases before the cluster were associated with an increased risk. Neither the detection of *Legionella* in the water system nor the type of disinfection were found to be associated with the risk of a new case. Small accommodations (< 36 rooms) had a lower risk of occurrence of further cases compared with larger ones.

The TALD surveillance scheme operated by ELDSNet is unique and provides valuable data on accommodations associated with TALD cases. From 1988–2005 there was a continuous increase in the number of TALD cases reported to ELDSNet; however, this number plateaued between 2006–14, suggesting that the scheme had reached maturity [13]. This is of importance because it means that a large number of accommodations associated with TALD cases were included in our dataset during these years, increasing the system’s ability to detect accommodations that were associated with previous cases. Most of the accommodations included in the analysis were located in countries with a high number of visitors from countries that report TALD cases to ELDSNet [13,18]. This means that under-reporting of TALD cases that stayed in the accommodations included in this analysis is possible but unlikely.

The main limitations of our analysis are related to data quality issues. The surveillance scheme and its data collection were designed for operational purposes (i.e. detection and notification of clusters and follow-up of control measures). Countries report TALD cases to the European Surveillance System (TESSy) database, but notification and follow-up are monitored on the EPIS platform, which does not have the structure of a surveillance database. This means that there were no mandatory fields or validation rules on the final report on environmental investigation (form B) and, therefore, there were numerous missing values. In addition, some values collected as free text could not be included for the purpose of this analysis. For example, it is possible to report information on levels of *Legionella* in the water system (colony-forming unit (CFU)/L), but these values are collected as free text with poor data completeness and quality issues. We collected additional information on accommodation characteristics (number of rooms) through other data sources, but we could not capture other potentially relevant information such as the presence of a pool or a spa. For example, the largest cluster included in our analysis (15 cases) was associated with a hotel in Calpe, Spain in 2011–12 [19]. Genomic investigation of the outbreak later revealed that a spa-pool was the main source of infection and that the hotel might have been colonised by *Legionella* since its construction [20]. Last, our data were unable to capture changes in accommodation site characteristics over time (e.g. extension or new facilities).

The high proportion of accommodation sites that were associated with further cases questions the effectiveness of control measures and/or the way that these measures are reported. A previous study suggested that thermal disinfection alone would not be efficacious enough to eliminate *Legionella*, unless applied with other measures [21]. It is also documented that the *Legionella*-amoeba association could reduce the effectiveness of the treatments applied [22]. Yet, in our study we only captured cleaning by disinfection. The 26 accommodations that were not treated by disinfection may have undergone another type of cleaning (e.g. physical). In this study, we could not entirely control for a possible confounding by indication [23]. Accommodations that were deemed more at risk for a further case may have been selected for more intensive disinfection. We partly prevented this bias with the information on the preventive measures. Accommodations with preventive measures in place before the investigation were probably less likely to be selected for intensive disinfection.

It is hardly surprising that environmental investigations found *Legionella* in the water system of a large proportion of accommodation sites (ca 65%). Although we had no information on the laboratory tests used to detect *Legionella* in environmental samples, this high proportion suggests that the types of tests used would probably have little impact on our results. A study carried out in Italy showed that *Legionella pneumophila* was present in 74% of the hotels investigated by real-time PCR [24]. However, it is likely that only a fraction of these environmental isolates were *Legionella* strains associated with LD cases. A large study performed in France suggested that *Legionella pneumophila* serogroup 1 accounted for as much as 95% of clinical isolates positive for *Legionella*, but less than 30% of environmental isolates [25]. It is thought that only a subset of *Legionella* strains recovered from the environment cause LD in humans [7]. Unfortunately, such detailed information on strain type was not available for the purpose of this analysis. The vast majority of TALD cases are laboratory confirmed by urinary antigen test [13], which does not detect all *Legionella pneumophila* serogroups and does not allow for further strain characterisation. It is therefore not possible to match clinical and environmental findings.

There are possible factors that could explain the lower risk observed in small accommodations. First, a small number of rooms is a proxy for a small number of visitors. Since LD has a very low attack rate, a limited number of people exposed would yield a low number of cases, regardless of the risk. The second possible explanation is that small accommodations also have simpler water systems, which are easier to maintain. Last, small accommodations may be less likely to have a large proportion of their rooms unoccupied, because this may put them out of business.

In our analysis, the risk of recurrence decreased after 2 years, which suggests that recurrent cases may have been exposed to the same source as the cluster cases were. The small increase after 3 years relied on few observations and is therefore difficult to interpret. Yet, a previous case before the cluster—often years before the cluster—was a major factor associated with the occurrence of a further case after the cluster. Other studies reported cases associated with the same accommodation over a long period, including an investigation documenting LD cases associated with a hotel over a 20-year period [26]. Apart from control measures carried out in an unsatisfactory manner, Ricketts et al. listed a few possible explanations for recurrent cases from their previous analyses [6], including new staff not properly trained in control procedures, closure and reopening without rigorous application of control measures and complex water systems (e.g. dead legs of pipework). Our analysis could not explore the impact of such possible factors.

In the subanalysis restricted to accommodations with no previous cases, accommodations associated with a cluster of three or more cases had a lower risk for a subsequent case compared with those associated with a cluster of only two cases, regardless of the size of the accommodation. This may suggest that public health authorities carry out stricter and/or more comprehensive control measures when facing large clusters of TALD cases, and/or that being associated with such clusters provides a strong incentive to hotel owners to maintain prevention measures in the long term.

### Conclusion

In conclusion, accommodation size and multiple earlier LD cases were predictive of further LD cases.

TALD cluster sites previously associated with TALD cases should receive special attention and possibly scaled-up control measures. Further, the surveillance scheme would benefit from more integration of the data collection systems and the standardised collection of key variables, such as the level of *Legionella* in the water system or information on causative *Legionella* strains using molecular data. The scheme would also benefit from capturing information on implemented preventive and control measures at a more detailed level.

## References

[1] Fields BS Benson RF Besser RE Legionella and Legionnaires’ disease: 25 years of investigation. Clin Microbiol Rev. 2002;15(3):506-26. 10.1128/CMR.15.3.506-526.2002 12097254 PMC118082

[2] Correia AM Ferreira JS Borges V Nunes A Gomes B Capucho R Probable Person-to-Person Transmission of Legionnaires’ Disease. N Engl J Med. 2016;374(5):497-8. 10.1056/NEJMc1505356 26840151

[3] Beauté J The European Legionnaires’ Disease Surveillance Network Legionnaires’ disease in Europe, 2011 to 2015. Euro Surveill. 2017;22(27):30566. 10.2807/1560-7917.ES.2017.22.27.30566 28703097 PMC5508329

[4] Fraser DW Tsai TR Orenstein W Parkin WE Beecham HJ Sharrar RG Legionnaires’ disease: description of an epidemic of pneumonia. N Engl J Med. 1977;297(22):1189-97. 10.1056/NEJM197712012972201 335244

[5] Grist NR Reid D Najera R Legionnaires’ disease and the traveller. Ann Intern Med. 1979;90(4):563-4. 10.7326/0003-4819-90-4-563 434635

[6] Ricketts KD Yadav R Rota MC Joseph CA European Working Group for Legionella Infections Characteristics of reoffending accommodation sites in Europe with clusters of Legionnaires disease, 2003-2007. Euro Surveill. 2010;15(40):19680. 10.2807/ese.15.40.19680-en 20946756

[7] Phin N Parry-Ford F Harrison T Stagg HR Zhang N Kumar K Epidemiology and clinical management of Legionnaires’ disease. Lancet Infect Dis. 2014;14(10):1011-21. 10.1016/S1473-3099(14)70713-3 24970283

[8] Ricketts K Joseph C European Working Group for Legionella Infections Travel-associated Legionnaires’ disease in Europe: 2003. Euro Surveill. 2004;9(10):480. 10.2807/esm.09.10.00480-en 29183473

[9] European Centre for Disease Prevention Control (ECDC). Legionnaires' disease in Europe, 2011. ECDC surveillance report. Stockholm: ECDC; 2013.

[10] European Centre for Disease Prevention Control (ECDC). Legionnaires' disease in Europe, 2012. ECDC surveillance report. Stockholm: ECDC; 2014.

[11] European Centre for Disease Prevention Control (ECDC). Legionnaires' disease in Europe, 2013. ECDC surveillance report. Stockholm: ECDC; 2015.

[12] European Centre for Disease Prevention and Control (ECDC). Legionnaires' disease in Europe, 2014. ECDC surveillance report. Stockholm: ECDC; 2016.

[13] European Centre for Disease Prevention and Control (ECDC). Legionnaires' disease in Europe, 2015. ECDC surveillance report. Stockholm: ECDC; 2017.

[14] European Centre for Disease Prevention and Control (ECDC). European Legionnaires' Disease Surveillance Network (ELDSNet): operating procedures. ECDC technical document. Stockholm: ECDC; 2012.

[15] European Commission. 2012/506/EU: Commission Implementing Decision of 8 August 2012 amending Decision 2002/253/EC laying down case definitions for reporting communicable diseases to the Community network under Decision No 2119/98/EC of the European Parliament and of the Council. Official Journal of the European Union. Luxembourg: Publications Office of the European Union. 27.9.2012:L 262/1. Available from: https://eur-lex.europa.eu/legal-content/EN/TXT/?uri=CELEX%3A32012D0506

[16] Ricketts KD Slaymaker E Verlander NQ Joseph CA What is the probability of successive cases of Legionnaires’ disease occurring in European hotels? Int J Epidemiol. 2006;35(2):354-60. 10.1093/ije/dyi317 16434431

[17] Bradburn MJ Clark TG Love SB Altman DG Survival analysis Part III: multivariate data analysis -- choosing a model and assessing its adequacy and fit. Br J Cancer. 2003;89(4):605-11. 10.1038/sj.bjc.6601120 12915864 PMC2376927

[18] Beauté J Zucs P de Jong B Risk for travel-associated legionnaires’ disease, Europe, 2009. Emerg Infect Dis. 2012;18(11):1811-6. 10.3201/eid1811.120496 23092591 PMC3559146

[19] Vanaclocha H Guiral S Morera V Calatayud MA Castellanos M Moya V Preliminary report: outbreak of Legionnaires disease in a hotel in Calp, Spain, update on 22 February 2012. Euro Surveill. 2012;17(8):20093. 22401506

[20] Sánchez-Busó L Guiral S Crespi S Moya V Camaró ML Olmos MP Genomic Investigation of a Legionellosis Outbreak in a Persistently Colonized Hotel. Front Microbiol. 2016;6:1556. 10.3389/fmicb.2015.01556 26834713 PMC4720873

[21] Mouchtouri V Velonakis E Hadjichristodoulou C Thermal disinfection of hotels, hospitals, and athletic venues hot water distribution systems contaminated by Legionella species. Am J Infect Control. 2007;35(9):623-7. 10.1016/j.ajic.2007.01.002 17980243

[22] Cervero-Aragó S Rodríguez-Martínez S Puertas-Bennasar A Araujo RM Effect of Common Drinking Water Disinfectants, Chlorine and Heat, on Free Legionella and Amoebae-Associated Legionella. PLoS One. 2015;10(8):e0134726. 10.1371/journal.pone.0134726 26241039 PMC4524690

[23] Rothman KJ, Greenland S, Lash TL. Modern epidemiology. 3rd ed. ed. Philadelphia: Lippincott Williams & Wilkins; 2008.

[24] Bonetta S Bonetta S Ferretti E Balocco F Carraro E Evaluation of Legionella pneumophila contamination in Italian hotel water systems by quantitative real-time PCR and culture methods. J Appl Microbiol. 2010;108(5):1576-83. 10.1111/j.1365-2672.2009.04553.x 19796090

[25] Doleans A Aurell H Reyrolle M Lina G Freney J Vandenesch F Clinical and environmental distributions of Legionella strains in France are different. J Clin Microbiol. 2004;42(1):458-60. 10.1128/JCM.42.1.458-460.2004 14715805 PMC321724

[26] Cowgill KD Lucas CE Benson RF Chamany S Brown EW Fields BS Recurrence of legionnaires disease at a hotel in the United States Virgin Islands over a 20-year period. Clin Infect Dis. 2005;40(8):1205-7. 10.1086/428844 15791524

